# Retrospective analysis of HIV-associated lymphomas: insights from a single Romanian center over 15 years

**DOI:** 10.3389/fonc.2025.1569433

**Published:** 2025-06-18

**Authors:** Monica-Daniela Padurariu-Covit, Iulia Chiscop, Cristian Gutu, Anca-Adriana Arbune, Elena Niculet, Manuela Arbune

**Affiliations:** ^1^ Doctoral School of Biomedical Sciences, “Dunarea de Jos” University, Galati, Romania; ^2^ Hematology Department, “Sf. Apostol Andrei” Emergency County Hospital, Galati, Romania; ^3^ Clinical Surgical Department, “Dunarea de Jos” University from Galati, Galati, Romania; ^4^ Clinical Medical Department, Faculty of Medicine and Pharmacy, “Dunarea de Jos” University of Galati, Galati, Romania; ^5^ Neurology Department, Fundeni Clinical Institute, Bucharest, Romania; ^6^ Multidisciplinary Integrated Center for Dermatological Interface Research, “Dunărea de Jos” University, Galati, Romania; ^7^ Morphological and Functional Sciences Department, “Dunarea de Jos University”, Galati, Romania; ^8^ Pathology Department, “Sf. Apostol Andrei” Emergency County Hospital, Galati, Romania; ^9^ Clinical Medical Department, “Dunarea de Jos University”, Galati, Romania; ^10^ Clinical Hospital for Infectious Diseases, “Sf. Cuv. Parascheva”, Galati, Romania

**Keywords:** HIV, AIDS, lymphoma, antiretroviral treatment, opportunistic infections, non-AIDS co-morbidities

## Abstract

**Introduction:**

Lymphoma is a significant cause of mortality among people living with human immunodeficiency virus (PLWH). The objective of our study was to assess the characteristics of lymphomas in PLWH in a single center from the southeast of Romania.

**Methods:**

We retrospectively analyzed the prevalence and clinical and demographic characteristics of patients with lymphoma associated with HIV/AIDS monitored over a period of 15 years. Kaplan–Meier analysis was used to estimate survival rates and evaluate the risk of mortality in lymphoma patients.

**Results:**

Among the 476 new cases of HIV/AIDS registered, 9 cases of lymphoma were identified, representing a prevalence of 1.89%. Overall mortality was 13.6%, with lymphoma contributing to 10.76% of HIV/AIDS-related deaths. The average age at lymphoma diagnosis was 37 years, with most patients being men and smokers with sexually transmitted HIV. Common coinfections included hepatitis B virus (HBV) and tuberculosis. Advanced-stage disease (Ann Arbor stage IV) and type B clinical symptoms were present in half of the cases. Oncological treatment was provided in 5 cases, achieving a survival rate of 30%.

**Conclusions:**

The high mortality highlights the need for early diagnosis and an integrated therapeutic approach to improve the prognosis of patients with HIV-associated lymphomas.

## 1 Introduction

Globally, the epidemic of the human immunodeficiency virus (HIV) is ongoing, with an estimated 39.9 million people living with HIV (PLWH) (1).

Nowadays, due to the huge scientific advances in the understanding of the viral pathways, as well as their diagnosis, treatment, and prevention, HIV has become a chronic, manageable disease, with a longer life expectancy. However, despite the advancements in public health strategies and treatment, a definitive cure for HIV is not yet available. The persistent viral inflammation in PLWH is related to frailty, in terms of precocious aging and frequent additional chronic conditions, such as cardiovascular, kidney, and bone diseases and various cancers (2).

HIV-positive people have a higher risk of developing various diseases, including cancer. Progressive immunosuppression secondary to HIV infection is a risk factor for the development of a variety of malignancies (3, 4).

The prolonged suppression of HIV replication with undetectable blood viral load under effective antiretroviral therapies (ARTs) has decreased the opportunistic infections and neoplasms associated with severe acquired immunodeficiency syndrome (AIDS).

The pathogenesis of HIV/AIDS-associated lymphoma is multifactorial, involving immune dysregulation, genetic mutations, viral coinfections, and chronic activation of B lymphocytes (2). These lymphomas predominantly originate from B cells and exhibit clonal immunoglobulin rearrangements (5).

Co-infections with HTLV-1, EBV, and HHV8 are oncogenic viruses that increase the susceptibility of PLWH to developing HIV/AIDS-associated lymphomas (5–7). An elevated risk of lymphomas was reported in HIV-positive individuals with HBV and HCV coinfections (8).

As an AIDS indicator, non-Hodgkin lymphoma (NHL) has continued to be the most common type of cancer and a leading cause of mortality in PLWH (9). Furthermore, Hodgkin lymphoma (HL) is not classified as an AIDS-defining illness, but studies have reported an increased incidence of HL in PLWH compared to the general population (10).

NHL is the 12th most common cancer worldwide, with a global incidence of 5.6 cases per 100,000 people in 2022, while HL ranks 26th and has a much lower incidence of 0.95 cases per 100,000 people (11). The incidence of NHL and HL in people living with HIV (PLWH) was 10- to 20-fold higher compared to the general population, but the clinical features and prognostic factors remain poorly differentiated from non-HIV lymphoma (12, 13).

Some types of lymphomas, such as primary diffuse large B-cell lymphoma or primary cerebral lymphoma, are opportunistic diseases considered as indicators for the classification in the AIDS stage (14, 15).

Regarding AIDS-associated lymphomas, it is estimated that more than 40% of patients in the advanced stages of immunosuppression could be diagnosed with one of these hematologic malignancies (16).

In developed countries, NHL is the most common cause of death associated with HIV infection, accounting for 23% to 30% of all AIDS-related deaths (16). HIV-related NHLs occur in patients with advanced HIV infection with a T-lymphocyte (CD4^+^) count of less than 100 cells/μL and a high HIV viral load (17).

HL is the most common type of cancer in HIV-positive patients, not associated with AIDS. HL in non-HIV patients had a bimodal age distribution with an initial peak at 20–30 years and a second peak at 50–65 years, while the mean age of presentation of HL in HIV-positive patients was 41 years in European countries and 34 years in African countries (18, 19). Globally, 0.4% of new cancer cases and 0.2% of cancer-related deaths were due to HL in 2020 (20). The incidence of HL varied by sex, age, and geographic location. People at a higher risk for HL included men, adolescents, young adults, and those with a history of Epstein–Barr virus infection, HIV/AIDS, autoimmune diseases, exposure to pollution, and smoking as well as family history (21–23).

The most common histologic types of HIV-associated lymphomas were diffuse large B-cell lymphoma (DLBCL; 37%), HL (26%), and Burkitt lymphoma (BL; 20%). Low CD4^+^ T-cell counts and HIV-1 viral replication (VL) are independent risk factors for DLBCL in people living with HIV (24). Other types, such as primary effusion lymphoma (PEL), primary central nervous system lymphoma (PCNSL), and plasmablastic lymphoma (PBL), were less common (24, 25).

The GLOBOCAN database, designed by the International Agency for Research on Cancer (IARC), contains projected national cancer estimates up to 2024 derived from the best available recorded data from national (or subnational) cancer registries and national centralized registry systems in 185 countries or territories around the world (11, 26, 27).

The GLOBOCAN database provides comprehensive global data on cancer epidemiology, including lymphomas (12). However, while significant data exist on HIV-associated lymphomas globally (28), their epidemiology and clinical profiles in Romania remain poorly understood, necessitating localized studies.

Therefore, the present study provides a comprehensive analysis of the frequency, clinical features, and mortality associated with HIV/AIDS-related lymphomas in a single-center cohort from Romania. By focusing on a population characterized by unique epidemiological and clinical features, including pediatric HIV cohort survivors, the study highlights challenges such as advanced disease stages at diagnosis, high mortality rates, and limited access to innovative therapies like CAR-T. These findings emphasize the urgent need for personalized management strategies and underline the importance of addressing regional challenges in the care of patients with HIV and lymphoma.

## 2 Materials and methods

### 2.1 Study design and statistical analysis

This study is an observational, descriptive, and retrospective analysis, which aims to evaluate the survival outcomes of patients diagnosed with lymphoma in the context of HIV/AIDS. We performed a comparative analysis using Kaplan–Meier (K-M) survival curves to assess the prognosis of these patients over a period of 15 years (2008–2022). To conduct the statistical survival analysis, we divided the patients into two groups: one group consisting of patients with HIV/AIDS and lymphoma versus another group with HIV/AIDS only.

Patients with incomplete or missing medical records, especially regarding HIV diagnosis or histological confirmation of lymphoma, were excluded from the study to ensure the accuracy of the data.

The HIV/AIDS database was obtained from the Infectious Diseases Clinic Hospital Galati electronic database, which is a single center for PLWH in the Galati district (29–33). Our center was located on the southeast border of Romania, providing healthcare for PLWH, with an estimated general population of 600,000 people.

We selected the diagnostic-related group codes B.20–24, from 1 January 2008 to 31 December 2022 (34). Additionally, we have identified the HIV/AIDS patients with lymphomas, by the diagnostic code B.21.1, B.21.2, or B.21.3, specifying HIV disease resulting in Burkitt lymphoma, non-Hodgkin lymphoma, or other malignant neoplasms of the lymphoid, hematopoietic, and related tissue (34) All the cases were revised in December 2023, covering at least 1 year of HIV/AIDS follow-up. The cases were categorized as follows: retained in care (patients continuing to be monitored and treated in the center), deaths, and lost to follow-up (patients with no updated information). The endpoint of the study was either death or 31 December 2022. The frequency of lymphomas in PLWH was calculated by dividing the number of HIV/AIDS and lymphoma cases by the total number of HIV/AIDS diagnosed cases during the study time. Mortality was calculated among patients with HIV/AIDS and lymphomas and was compared with mortality among the cases with HIV/AIDS only.

To achieve the second objective, we have selected cases with HIV/AIDS and lymphoma from our institution’s archived records, and we have studied their detailed medical files.

### 2.2 HIV and lymphoma patient overview

We analyzed demographic data, HIV history, and lymphoma history. We examined whether the lymphoma diagnosis was an indicator of immunosuppression and a reason for HIV testing. HIV history comprised the pattern of transmission, age and year of diagnosis, staging of HIV diagnosis, coinfections, nadir of CD4 count, and antiretroviral treatment (ART) experience. The stages of HIV infection were evaluated using the CDC classification system, considering the immunological level based on CD4 count and the clinical criteria for AIDS-related conditions (35). The nadir of CD4 count means the lowest ever count in an individual with HIV. Antiretroviral therapy was used according to the timeline versions of European guidelines, combining nucleoside(tide) reverse transcriptase inhibitor (NRTI), non-nucleoside reverse transcriptase inhibitor (NNRTI), protease inhibitor (PI), or integrase inhibitor class (II) (36).

### 2.3 Lymphoma staging and clinical features

In the characterization of lymphoma, the year of diagnosis, concomitant CD4 count, staging of lymphoma, histopathology, oncologic treatment, major complications, and comorbidities were considered. The diagnosis of lymphoma was based on an anatomical-pathological examination supplemented by immunohistochemistry, performed on various relevant tissues, including lymph nodes, skin or mucous membranes, and bone marrow. The lymphomas were staged according to the Ann Arbor classification system. The Ann Arbor staging system classifies HL and NHL by disease extent: stage I (single lymph node region), stage II (multiple lymph node regions on the same side of the diaphragm), stage III (lymph nodes on both sides of the diaphragm), and stage IV (diffuse or disseminated involvement of one or more extra lymphatic organs, or either involvement of the liver, bone marrow, or lungs). Each stage is subdivided into A (no systemic symptoms), B (systemic symptoms—fever exceeding 38°C without a known cause, severe night sweats, or weight loss greater than 10% of body weight within 6 months before diagnosis), E (spread of lymphoma to organs outside the lymphatic system, such as the liver, bone marrow, or lungs), S (spleen involvement), and bulky disease [for HL, bulk is defined as any single node or nodal mass with a diameter ≥10 cm or mediastinal mass ratio (maximum width of mediastinal mass/maximum intrathoracic diameter >0.33); for DLBCL, bulky disease means any nodal or extra nodal tumor mass with a diameter of ≥7.5 cm] (37–39).

### 2.4 Statistical analysis and survival assessment

To analyze data, the K-M survival analysis was employed. To conduct the univariate approach of K-M, we divided the patients into two groups: one group consisting of patients with HIV/AIDS and lymphoma and another group with HIV/AIDS only. Incomplete observations of patients such as those lost to follow-up or without an event occurring by the end of the study were censored, meaning their data were considered incomplete in the survival analysis. The log-rank test was used to compare the survival estimates between groups, considering a *p*-value lower than 0.05 as significant. We used the SPSS software package version 26 for the Kaplan–Meier survival curve analysis of both groups. We analyzed the characteristics of patients with HIV/AIDS and HL or NHL using the two-sample test due to a small sample size.

## 3 Results

### 3.1 The frequency and mortality of HIV/AIDS-associated lymphomas

From 2008 to 2022, 476 PLWH were monitored in our center. These 476 cases were divided into two groups: a group of 9 PLWH diagnosed with lymphoma (7 NHL and 2 HL) (**Table 1**) and a group of 467 PLWH with HIV/AIDS only, who did not develop lymphoma.

**Table 1:** Individual characteristics of PLWH diagnosed with lymphoma.

NHL								HL	
Patient ID	P1	P2	P3	P4	P5	P6	P7	P8	P9
Gender	Male	Male	Male	Male	Male	Male	Female	Male	Male
Age HIV dg	10	48	20	21	2	59	35	16	57
Age L dg	18	50	21	21	26	59	35	28	57
Living area	Urban	Urban	Urban	Rural	Rural	Urban	Urban	Rural	Rural
Education#	8	12+	4	8	8	12	12	12	8
Marital status	Single	Married	Single	Single	Single	Divorced	Single	Single	Widower
Smoking	No	Yes	No	Yes	Yes	Yes	Yes	No	Yes
HIV-related data									
Year HIV dg	1999	2006	2009	2013	1992	2020	2021	2002	2021
Transmission pattern	Pediatric	Sexual	Pediatric	Sexual	Pediatric	Sexual	Sexual	Pediatric	Sexual
HIV staging^a^	B2	C3	C3	C3	B2	C3	C3	B3	C3
AIDS^a^	No	Yes	Yes	Yes	No	Yes	Yes	Yes	Yes
Nadir CD4 mm^3^	47	28	4	15	20	66	58	31	21
No. of ART lines	5	2	2	1	6	1	1	3	1
TB history	Yes	Yes	Yes	No	Yes	No	No	Yes	No
Syphilis history	No	No	No	No	No	No	No	No	No
HBV coinfection	Yes	No	Yes	No	Yes	No	No	Yes	No
HCV	No	No	No	No	No	No	No	No	No
Lymphoma-related data									
Year L dg	2008	2008	2010	2013	2017	2020	2021	2017	2021
CD4 on L dg mm^3^	165	172	74	15	85	66	58	110	112
B symptoms	No	No	No	Yes	Yes	No	No	Yes	Yes
L staging	III	IV	IV	IV	III	IV	IV	IV	III
Biopsy-HPE	LN	Skin	Skin	LN	LN	Parotid	Maxillary	BM	LN
IHC	Yes	Yes	Yes	Yes	No	Yes	Yes	Yes	Yes
L subtype	DLBCL	SLL	BL	DLBCL	DLBCL	DLBCL GCB	PBL	NSCHL	MCHL
Major Complications	Myasthenia gravis	Nephrolithiasis seizures Retinal detachment in the right eye	Liver failure UGIB	Tetraparesis UGIB	Liver failure UGIB		Sepsis Left breast carcinoma	Sepsis	MSOF
COVID-19	–	–	–	–	–	No	Yes	–	Yes
Other Comorbidities		Plasmacytoma Diabetes	Mental deficiency				Psoriasis	Warts	HBP Pleurisy
Oncology treatment	5 CHOP	6 CVP	No	No	No	8 RCHOP	3 EPOCH	6 ABVD	No
Survive (a)	<1	<1	<1	<1	<1	>2	<1	>6	<1
Survive (b)	9	2	1	<1	24	>2	<1	>21	<1

ABVD, doxorubicin, bleomycin, vinblastine, and dacarbazine; ART, antiretroviral treatment; BL, Burkitt lymphoma; BM, bone marrow; CHOP, cyclophosphamide, doxorubicin, vincristine, and prednisone; CVP, cyclophosphamide, doxorubicin, vincristine; D, divorced; dg, diagnostic; DLBCL, diffuse large B-cell lymphoma; Education#, years of formal education; EPOCH, etoposide, prednisone, vincristine, cyclophosphamide, doxorubicin; GCB, germinal center B cell; HBV, hepatitis B virus; HCV, hepatitis C virus; HL, Hodgkin lymphoma; HPE, histopathology examination; IHC, immunohistochemistry; L, lymphoma; LN, lymph nodes; MCHL, mixed cellularity Hodgkin lymphoma; MODS, multiple organ dysfunction syndrome; NHL, non-Hodgkin lymphoma; NSCHL, nodular sclerosis classic Hodgkin lymphoma; PBL, plasmablastic lymphoma; R-CHOP, rituximab, cyclophosphamide, doxorubicin, vincristine and prednisone; SLL, small lymphocytic lymphoma; Survive (a), survival years after the diagnosis of lymphoma; Survive (b), survival years after HIV diagnosis; UGIB, upper gastrointestinal bleeding.

a These data correspond to the staging at the time of HIV diagnosis, while the nadir represents the lowest recorded CD4 count from the time of HIV diagnosis until the diagnosis of lymphoma. Although antiretroviral therapy was available, some patients continued to experience immunological decline after HIV diagnosis, potentially contributing to the development of malignant hematological disorders.

Of the total 476 PLWH, 366 were alive, 65 died, and 45 were lost to follow-up. Thus, the overall death rate was 15.08%. The 45 cases lost to follow-up were not excluded from the analysis.

Among the 476 cases of PLWH, we have identified 9 cases with lymphomas, specified as 7 NHL and 2 HL, meaning a 1.89% (9/476 × 100) prevalence of lymphoma comorbidity. Of the 65 total deaths reported in the study, 7 were PLWH diagnosed with lymphomas, accounting for 10.76% of all deaths. The mortality rate of HIV/AIDS-associated lymphomas was 77.7% (7/9 × 100), compared with 12.41% (58/467 × 100) deaths in the HIV/AIDS-only group.

The analysis of K-M curves identified a chi-square value of 13,456 (*df* = 1), and an associated *p*-value (Sig.) <0.001, indicating that PLWH diagnosed with lymphoma had a significantly shorter life expectancy compared to those with HIV/AIDS only.

**Figure 1:**
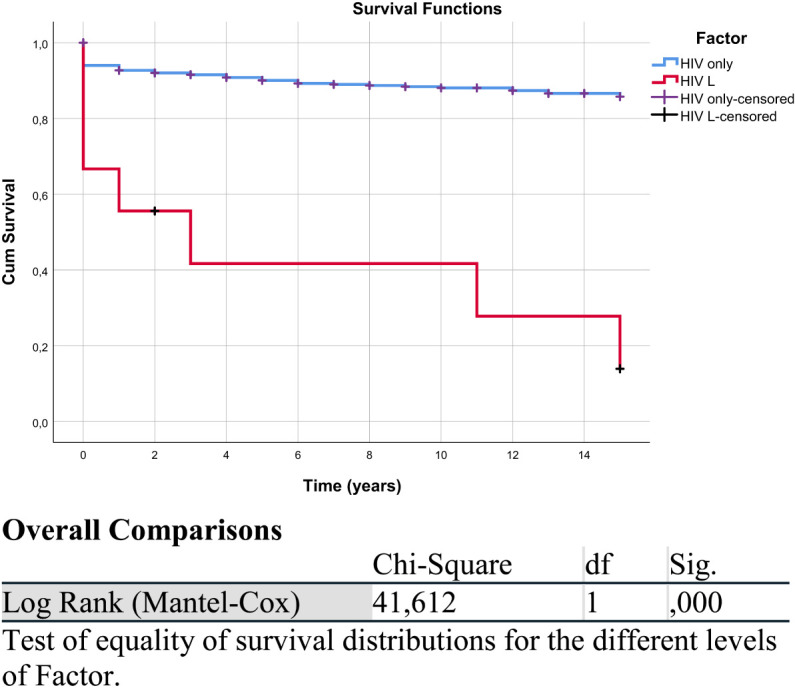
Kaplan–Meier survival curve of the HIV/AIDS and lymphoma (HIV L) group compared with the HIV/AIDS-only group. “HIV only-censored” and “HIV L-censored” refer to patients in the respective groups (HIV without lymphoma and HIV with lymphoma) whose data were censored, meaning they did not experience the event of interest, defined here as overall survival (death from any cause), or were lost to follow-up. The overall comparison table corresponds to this figure.

The analysis of Kaplan–Meier curves identified a statistically significant difference in survival distributions between the two groups (chi-square = 13.456, *df* = 1, *p* < 0.001), indicating that PLWH diagnosed with lymphoma had a significantly shorter life expectancy compared to those with HIV/AIDS only (**Figure 1**, **Table 2**).

**Table 2:** Means and medians for survival time (years).

Factor	Mean^a^				Median			
	Estimate	Std. error	95% confidence interval		Estimate	Std. error	95% confidence interval	
			Lower bound	Upper bound			Lower bound	Upper bound
HIV only	13.432	.204	13.031	13.832	.	.	.	.
HIV L	6.222	2.423	1.473	10.971	3.000	2.490	.000	7.880
Overall	13.296	.209	12.885	13.706	.	.	.	.

a Estimation is limited to the largest survival time if it is censored.

### 3.2 Characteristics of PLWH diagnosed with lymphomas

#### 3.2.1 Demographic characteristics

The demographic characteristics described in this section apply to the PLWH cases diagnosed with lymphoma. Among the nine cases, seven were diagnosed with NHL and two with Hodgkin lymphoma (HL). All patients were Caucasian, most of them were men (eight out of nine) living in an urban area (five out of nine), and all but one had completed secondary education. According to marital status, most were single (six out of nine), one case was divorced, one was married, and one was widowed. The age at HIV diagnosis for the nine PLWH diagnosed with lymphoma ranged between 2 and 59 years, including four patients with a pediatric pattern of HIV infection and five sexually transmitted cases (**Table 1**). None of the patients were intravenous drug users. The average age of lymphoma diagnosis was 35 ± 16.18, varying between 18 and 60 years. The diagnostic age of lymphomas was lower in smokers and patients with a pediatric pattern of transmission, history of tuberculosis, and hepatitis B virus (HBV) coinfection (**Table 3**).

**Table 3:** Factors influencing the age of lymphoma diagnosis in PLWH (two-sample *t*-tests).

*N* = 9		*n*	Average age ± SD	CI- 0.95	*p*
Lymphoma subtype	NHL	7	32.85 ± 15.97	−209.21; 189.92	0.649
	HL	2	42.5 ± 20.50		
Smoking	Yes	6	41.33 ± 16.25	1.21; 36.78	*0.039*
	No	3	22.33 ± 5.13		
Pattern of HIV transmission	Pediatric	4	23.25 ± 4.57	−42.14; −0.15	*0.048*
	Sexual	5	44.4 ± 16.11		
Tuberculosis history	Yes	5	28.6 ± 12.60	−41.97; 13.17	0.237
	No	4	43 ± 18.25		
HBV-coinfection	Yes	4	23.25 ± 4.57	−42.14; −0.15	*0.048*
	No	5	44.4 ± 16.11		

The values in italics are used to highlight the p-values that are less than 0.05.

#### 3.2.2 HIV history characteristics

On HIV diagnosis, seven out of nine patients were late presenters (AIDS stage), while immunosuppression had already progressed in the remaining two patients. As a result, all of them experienced a very low average nadir CD4 count of 32.22 ± 20.65, ranging between 4 and 66/mm^3^ (**Table 1**). A history of tuberculosis was found in five out of the nine cases, and four out of the nine cases had both HBV coinfection and tuberculosis. There was no coinfection with hepatitis C virus (HCV) or syphilis. All patients have experienced at least one line of ART after HIV diagnosis, but three of them had multiple ART treatments. Baseline HIV viral load was variable, and undetectable levels under ART were temporarily achieved in only two cases (P2, P3).

#### 3.2.3 Characteristics of HIV/AIDS lymphoma cases

Lymphoma was an indicator of immunosuppression, as it is frequently observed in individuals with advanced HIV infection, where the immune system is significantly compromised. It was identified as an indicator for HIV diagnosis in four out of nine cases, all of them being “very late presenters” (patients P4, P6, P7, P9) (**Table 1**). The average CD4 count during the diagnosis of lymphoma was 95.22 ± 50.61, ranging from 15 to 172/mm^3^. In patient P3, the small lymphocytic lymphoma was interpreted as part of the immune reconstitution inflammatory syndrome (IRIS) following the diagnosis and treatment of tuberculosis coinfection and HIV. Specifically, the ART regimen of patient P3 included zidovudine + lamivudine + efavirenz and enfuvirtide. In the other cases (patients P1, P2, P5, and P8), the interval after HIV diagnosis to lymphoma occurrence ranged between 2 and 24 years (**Table 1**).

The diagnosis of lymphoma was confirmed by anatomopathological examinations of biopsy specimens of lymph node (4/9), skin or mucosa (4/9), and bone marrow (1/9). Immunohistochemistry was unavailable in one single case (P5).

The histological subtype of NHL was mostly DLBCL (44.44%). Other subtypes included small lymphocytic lymphoma, BL, and PBL, with one case for each. HL represented 22.22%. The subtypes were determined by microscopic evaluation and confirmation with specific immunohistochemical markers. The staging of lymphomas revealed invasion of extralymphatic tissues in six out of nine patients (P2, P3, P4, P6, P7, P8), which means stage IV according to Ann Arbor staging (11, 34). The other three patients (P1, P5, P9) were categorized as stage III. The signs of B category (33) were present in four out of nine patients: fever (P4, P5, P8, P9), over 10% of weight loss in the last 6 months (P4), and night sweats (P4) (**Table 1**).

Chemotherapy was provided to five patients (P1, P2, P6, P7, P8), and two of them had survived (P6, P8). The other three patients (P1, P2, P7) were non-responders to chemotherapy, and the lymphomas had progressed to a fatal outcome. P1 and P6 received cyclophosphamide, doxorubicin, vincristine, and prednisone treatment (CHOP), but P6 also received immunotherapy with rituximab (R), which may be an important factor in the patient’s profound response and survival (**Table 1**). The 1-year survival rate was 22.22%, and there were only two survivors: one case each of NHL and HL. The inability to administer chemotherapy in some cases was due to late presentation and the presence of active infections, such as COVID-19 (P9), as well as delays in obtaining immunohistochemical results.

One particular case was a female patient (P7) who had two types of cancers. The first one was an oral PBL, an indicator of HIV diagnosis, and the second was a tumor of the left breast that was detected 3 months later, during the chemotherapy for lymphoma. The breast carcinoma diagnosis was supported by the anatomopathological exam, but the result was available after the patient died.

## 4 Discussion

### 4.1 Pathogenesis of HIV/AIDS-associated lymphoma: mechanisms and risk factors

HIV-related chronic inflammation is associated with the disruption of cytokines and chemokines that play a role in the tumor microenvironment (40).

The mechanisms of enhanced death in HIV-infected cells versus uninfected ones are increased apoptosis, pyroptosis, and ferroptosis (41). The HIV-related CD4 T-cell death involves approximately a 95% caspase-1-mediated pyroptosis pattern, while the proportion of cell apoptosis is less than 5% (42). Significant differences in microenvironments were found between sporadic DLBCL and HIV-associated DLBCL, which was highly angiogenic with a higher density of microvessels (43). The type of lymphoma was significantly influenced by the degree of CD4 cell depletion and immune dysfunction (44).Patients with CD4^+^ T-cell depletion are more likely to develop aggressive subtypes such as DLBCL, PEL, or PBL. In contrast, those with higher CD4^+^ counts are prone to centroblastic DLBCL and BL (24).

However, HIV could influence oncogenesis independent of immunosuppression using a direct pro-oncogenic mechanism (45). The dysregulation of the cell cycle influences the non-immune microenvironment by increasing the extracellular matrix, profibrogenic factors, and aberrant lymphangiogenesis (46).

Chronic activation of B lymphocytes during HIV immune dysfunction leads to hypergammaglobulinemia, impaired humoral immunity, and germinal center hyperplasia, which can ultimately result in lymphoma development (47).

The degree of immune dysfunction, particularly reflected by CD4^+^ T-cell counts, plays a pivotal role in the clinical presentation, treatment response, and prognosis of HIV-associated lymphomas. Patients with severe immunosuppression, indicated by nadir CD4 counts below 50 cells/mm³, more frequently present with advanced-stage lymphomas (stages III/IV) and systemic symptoms, complicating management and worsening outcomes. Conversely, higher CD4 counts at lymphoma diagnosis correlate with better chemotherapy tolerance and longer survival, as observed in our cohort (48). For example, patient 1, with a CD4 count of 165 cells/mm³, exhibited a favorable response and prolonged survival, while patient 4, with a CD4 count of 15 cells/mm³, faced treatment-limiting complications.

Chronic immune activation driven by persistent HIV replication exacerbates immune dysfunction, depleting CD4^+^ T cells, disrupting immune surveillance, and fostering oncogenesis (49, 50).

These findings underline the necessity of integrating immune and virological monitoring into the diagnostic and therapeutic planning for HIV-associated lymphoma patients.

### 4.2 Epidemiology of HIV/AIDS and lymphoma co-occurrence

Recent updates from the United States (US) on HIV-associated lymphomas during the antiretroviral therapy indicate increasing incidence trends, with DLBCL being the most common subtype, followed by HL, concordant with the results of our study (13).

Some prospective cohort studies on different populations, conducted in Switzerland, the USA, or China, have reported the incidence of lymphomas in PLWH between 2% and 2.14% (51–53). We found an incidence of 1.89%, with a slight difference. It could possibly be explained by the younger HIV age demographics of patients in this study (HIV diagnosis age 2–57 years old). However, it is important to note that the prevalence of lymphomas increases significantly in patients diagnosed with AIDS, where more than 40% are affected by these malignancies (54). This highlights the critical need for early HIV diagnosis and management to prevent progression to advanced stages associated with a higher lymphoma burden (10).

Globally, malignancies, including lymphomas, are more common in men (55). The underlying reasons for this gender difference remain unclear but may involve extrinsic factors such as exposure to environmental carcinogens and intrinsic factors, comprising different immune and hormonal profiles, body size, and tumor biology in men and women (56). Our study confirms the predominance of lymphomas in men, most of them smokers, with a sexually transmitted pattern and severe immunosuppression. These demographic factors are commonly observed in other cohorts, and several studies suggest that smoking and advanced immunosuppression may contribute to a higher incidence and poor prognosis of lymphoma in HIV-infected individuals (46).

Lymphomas can develop across all age groups. Our study found that the average age at lymphoma diagnosis was 35 years, younger than that of other reports, such as 42 years in a Brazilian study, 48 years in a US study, or 43.6 years in a Chinese Study (18, 57, 58). The comparison may be influenced by the differences in sample size and age distribution across the studies.

Studies from Europe and the USA indicate that lymphomas are frequently diagnosed in patients with advanced immunosuppression, often as a late-stage complication in individuals with poorly controlled HIV (57). Furthermore, all our patients presented with advanced lymphomas (stage III/IV) and severe HIV immunosuppression (CD4 counts < 200/mm³). Although antiretroviral therapy was available, some patients continued to experience immunological decline after HIV diagnosis, potentially contributing to the development of malignant hematological disorders.

Studies in some European countries have identified a lower mortality rate of lymphoma among HIV patients. A French study for 10 years reported a mortality rate of 8.8% among 82,000 HIV patients (59). Another study conducted in the USA found a significantly higher mortality rate for HIV-associated lymphoma than for other HIV-related diseases (60). In Botswana, mortality in HIV HL was comparable to that of non-HIV HL, due to an equal increased access to oncologic care for PLWH and those without HIV (61).

The mortality rate of PLWH with lymphoma in our study was 77.7%, notably higher than the mortality rate of 13.65% in the HIV-only group. This stark difference underscores the severe impact of lymphoma on the survival of HIV Romanian patients.

### 4.3 Particularities of the Romanian HIV/AIDS epidemic and oncologic risk

Romania is a Central-Eastern European country with 18,359 alive PLWH reported by the Ministry of Health in June 2024 (62, 63). More than half of the cases are survivors of the “Romanian HIV pediatric cohort,” which means that patients were born mainly between 1988 and 1989. Most of them were infected with HIV during early childhood by a small amount of blood transfusions or inappropriate use of syringes and needles in healthcare institutions during the communist era. After the fall of communism in December 1989, the availability of HIV diagnosis tests revealed that Romania accounted for 60% of pediatric AIDS cases in Europe at that time (64).

The “HIV pediatric cohort” is characterized by its homogeneous Caucasian race, with many cases of long-term survival from early childhood to adulthood, predominantly of the HIV-1 subtype F, with a high rate of coinfection with hepatitis B virus and multiple antiretroviral drug experience (65). Growing up with HIV is a complex individual burden, involving physical, functional, psychological, and sociobehavioral development, putting one at high risk of increasing comorbidities. Three subjects with NHL (P1, P4, P5) and one with HL (P8) from our case series were part of this pediatric HIV cohort. They were diagnosed with HIV at the ages of 10 years, 19 years, 2 years, and 14 years, respectively, and developed lymphoma diagnosed at the ages of 18 years, 21 years, 26 years, and 29 years, respectively. Beyond the pediatric cohort (born in the 1990s), the “adult” HIV epidemic has progressed through increased sexual transmission or by intravenous drug use (66). A multicenter national database of oncologic HIV/AIDS-associated malignancies should be developed in Romania to better understand these rare comorbidities in our specific population and improve management strategies.

### 4.4 Immune reconstitution inflammatory syndrome

IRIS is a hyperinflammatory reaction that occurs when the immune system is recovering under antiretroviral therapy, mostly in PLWH with low CD4 counts and opportunistic infections with tuberculosis or cryptococcosis (67, 68). IRIS occurs in two forms: “paradoxical” IRIS is characterized by the exacerbation of a previously treated infection after ART is started, and “unmasking” IRIS is defined by the activation of a previously undiagnosed infection soon after antiretroviral therapy is started (69).

The “unmasking” IRIS was applied to one of our case series (P3). A study involving a cohort of 482 patients with HIV/AIDS-associated lymphoma from 1996 to 2011 found that 48 patients (10%) met the criteria for unmasking lymphoma. Among these cases, 10 (21%) were classified as HL, 19 (40%) as DLBCL, 4 (8%) as BL, 9 (19%) as PCNSL, and 6 (12%) as other NHL (70).

### 4.5 Real-life treatment, available options, prognosis, and future perspectives

The treatment of HIV infection and lymphoma requires an integrated approach, given the complex interplay between these two conditions. ARV is essential for the suppression of HIV replication and the recovery of immune function, while chemotherapy is the usual treatment of lymphoma with immunosuppressive adverse events (44).

Since 2018, the National Comprehensive Cancer Network (NCCN) guidelines have included dedicated recommendations for treating HIV-associated B-cell lymphomas (20, 71), highlighting the evolving approach to managing these co-occurring conditions in clinical practice.

The current standard practice is to continue or to initiate antiretrovirals during chemotherapy (66). PLWH who have well-controlled onco-hematologic disease have a life expectancy comparable to the general population. This group remains underrepresented in clinical trials, as seropositive status continues to be an exclusion criterion for most lymphoma trials (72, 73).

Therapeutic protocols such as R-CHOP (rituximab, cyclophosphamide, doxorubicin, vincristine, and prednisone), R-CDE (rituximab, cyclophosphamide, doxorubicin, etoposide), R-EPOCH, and (DA)-EPOCH-R (dose-adjusted etoposide, prednisone, vincristine, doxorubicin, and cyclophosphamide based on CD4 count plus rituximab) achieved a complete response rate of 69%–91%, with a 2-year survival rate of 62%–75% and low mortality from infectious causes. The addition of immunotherapy proved beneficial for patients with HIV-associated lymphomas (74).

The relationship between the number of chemotherapy cycles and prognosis in HIV-associated lymphomas offers valuable insights into treatment outcomes. In our cohort, patients underwent a range of regimens, including five cycles of CHOP, six cycles of CVP, eight cycles of R-CHOP, three cycles of EPOCH, and six cycles of ABVD. These data underline the importance of treatment intensity and duration in influencing overall survival (OS) and progression-free survival (PFS). Existing literature emphasizes that completing the full prescribed chemotherapy regimen is typically associated with higher rates of complete response and a reduced likelihood of recurrence. However, in HIV-positive patients, severe immunosuppression and associated complications can compromise treatment tolerance, often leading to premature discontinuation of chemotherapy. This underscores the need for personalized approaches to optimize both tolerability and efficacy in this unique patient population (75). The degree of immunosuppression, reflected in nadir CD4 counts and HIV staging, expresses the resources of immune recovery under ART and influences not only lymphoma aggressiveness but also the ability to complete treatment. Advanced immunodepression characterized all patients in our study with HIV-associated lymphomas, suggesting late presentation and delayed diagnosis of both HIV infection and lymphoma. This may reflect systemic deficiencies in screening and early detection within the Romanian healthcare system. Immune function markers such as CD4 counts are valuable prognostic indicators, as they reflect both the host’s capacity to tolerate chemotherapy and the biological aggressiveness of lymphoma in HIV-infected patients (76).

Autologous stem cell transplantation (ASCT) is provided equally for both HIV-negative and HIV-positive lymphoma patients from some European countries (77). In recent years, this therapeutic option has also been used in Romania for a few patients with HIV and lymphoma. The first two cases of HIV-associated DLBCL treated with the chimeric antigen receptor T-cell therapy (CAR-T) axicabtagene ciloleucel were reported in 2019. This report demonstrated that CAR-T cells can be successfully produced in HIV patients undergoing ART, even with CD4 counts as low as 52 cells/mm³ (78, 79). In Romania, CAR-T-cell therapy has been available since 2022 for adult patients with relapsed or refractory diffuse large B-cell lymphoma and for acute lymphoblastic leukemia who have failed at least two lines of systemic chemotherapy. However, this revolutionary treatment has not yet been administered to HIV-positive patients in our country.

### 4.6 Limitations of the study

The first limitation of our study is the retrospective design and the inconsistent availability of data. The evaluation and treatment decisions were not unitary because the guidelines and protocols for both HIV and lymphomas have been changed, considering the extended duration of the study. The HIV viral load was not systematically available due to either the temporary lack of reagents or the limited procedures caused by the COVID-19 pandemic. The limited population size diminishes the statistical power, and a study involving a larger patient cohort could yield more robust and precise conclusions in future research. Immunohistochemistry and other procedures for cancer diagnosis are missing because these are not covered by the health insurance and patients could not afford them. Serological evaluation of EBV and HHV-8 with oncogenic potential was not available.

## 5 Conclusions

Lymphoma is a rare comorbidity in PLWH from the South-East of Romania; however, it has a serious impact on life expectancy, due to the high mortality rate. The median age of PLWH with lymphoma was 35 years, ranging from 18 to 59 years old. The most frequent subtype of lymphoma was DLBCL. A low CD4 count is associated with more aggressive forms of lymphomas, with a low survival on 1-year follow-up. Smoking, HBV coinfection, and chronic infection with HIV of patients from “the pediatric cohort” are predictors for the younger age of lymphoma diagnosis. Most cases of lymphomas occurred in men with HIV/AIDS. Delay of the oncohematological diagnostic, the limited access to screening and treatment, and poor education for earlier medical visits are the main difficulties in the management of lymphomas in PLWH in Romania. These challenges highlight the need for sustained health strategies dedicated to this specific population.

## Data Availability

The original contributions presented in the study are included in the article/supplementary material. Further inquiries can be directed to the corresponding author.

## References

[1] WHO. The urgency of now: AIDS at a crossroads. Geneva: Joint United Nations Programme on HIV/AIDS (2024). Available at: https://www.unaids.org/sites/default/files/media_asset/2024-unaids-global-aids-update_en.pdf.

[2] Funderburg NT Huang SSY Cohen C Ailstock K Cummings M Lee JC. Changes to inflammatory markers during 5 years of viral suppression and during viral blips in people with HIV initiating different integrase inhibitor based regimens. Front Immunol. (2024) 15:1488799. doi: 10.3389/fimmu.2024.1488799 39600696 PMC11590120

[3] Landgren O Goedert JJ Rabkin CS Wilson WH Dunleavy K Kyle RA. Circulating serum free light chains as predictive markers of AIDS-related lymphoma. J Clin Oncol. (2010) 28:773–9. doi: 10.1200/JCO.2009.25.1322 PMC2834393 20048176

[4] Borgia M Dal Bo M Toffoli G. Role of virus-related chronic inflammation and mechanisms of cancer immune-suppression in pathogenesis and progression of hepatocellular carcinoma. Cancers. (2021) 13:4387. doi: 10.3390/cancers13174387 34503196 PMC8431318

[5] Carbone A Volpi CC Gualeni AV Gloghini A. Epstein–Barr virus associated lymphomas in people with HIV. Curr Opin HIV AIDS. (2017) 12:39–46. doi: 10.1097/COH.0000000000000333 27755151

[6] Noy A. Optimizing treatment of HIV-associated lymphoma. Blood. (2019) 134:1385–94. doi: 10.1182/blood-2018-01-791400 PMC7493463 30992269

[7] Rana I Dahlberg S Steinmaus C Zhang L. Benzene exposure and non-Hodgkin lymphoma: a systematic review and meta-analysis of human studies. Lancet Planetary Health. (2021) 5:e633–43. doi: 10.1016/S2542-5196(21)00149-2 PMC9109598 34450064

[8] Wang Q De Luca A Smith C Zangerle R Sambatakou H Bonnet F. Chronic hepatitis B and C virus infection and risk for non-Hodgkin lymphoma in HIV-infected patients: a cohort study. Ann Internal Med. (2017) 166:9–17. doi: 10.7326/M16-0240 27750294

[9] Berhan A Bayleyegn B Getaneh Z. HIV/AIDS associated lymphoma: review. Blood Lymphatic Cancer Targets Ther. (2022) 12:31–45. doi: 10.2147/BLCTT.S36132 PMC9063794 35517869

[10] Poizot-Martin I Lions C Allavena C Huleux T Bani-Sadr F Cheret A. Spectrum and incidence trends of AIDS- and non–AIDS-defining cancers between 2010 and 2015 in the french dat'AIDS cohort. Cancer Epidemiol Biomarkers Prev. (2021) 30:554–63. doi: 10.1158/1055-9965.EPI-20-1045 33310788

[11] Ferlay J Ervik M Lam F Laversanne M Colombet M Mery L. Global Cancer Observatory: Cancer Today. Lyon, France: International Agency for Research on Cancer (2024). Available at: https://gco.iarc.who.int/today.

[12] Kimani SM Painschab MS Horner MJ Muchengeti M Fedoriw Y Shiels MS. Epidemiology of haematological Malignancies in people living with HIV. Lancet HIV. (2020) 7:e641–651. doi: 10.1016/S2352-3018(20)30118-1 PMC10199168 32791045

[13] Wang C Xiao Q Zhang X Liu Y. HIV associated lymphoma: latest updates from 2023 ASH annual meeting. Exp Hematol Oncol. (2024) 13:65. doi: 10.1186/s40164-024-00530-6 38970132 PMC11225138

[14] Kaplan JE Benson C Holmes KK Brooks JT Pau A Masur H. Panel on Opportunistic Infections in Adults and Adolescents with HIV. In: Guidelines for the prevention and treatment of opportunistic infections in adults and adolescents with HIV: recommendations from the Centers for Disease Control and Prevention, the National Institutes of Health, and the HIV Medicine Association of the Infectious Diseases Society of America. (United States: NIH, CDC, IDSA). (2019). Available at: http://aidsinfo.nih.gov/contentfiles/lvguidelines/adult_oi.pdf.

[15] Horberg M Thompson M Agwu A Colasanti J Haddad M Jain M. Primary care guidance for providers of care for persons with human immunodeficiency virus: 2024 update by the HIV medicine association of the infectious diseases society of America. Clin Infect Dis. (2024) ciaa479. doi: 10.1093/cid/ciae479 39393187

[16] Re A Cattaneo C Rossi G. HIV and lymphoma: from epidemiology to clinical management. Mediterr J Hematol Infect Dis. (2019) 11:e2019004. doi: 10.4084/mjhid.2019.004 30671210 PMC6328036

[17] Chu Y Liu Y Fang X Jiang Y Ding M Ge X. The epidemiological patterns of non-Hodgkin lymphoma: global estimates of disease burden, risk factors, and temporal trends. Front Oncol. (2023) 13:1059914. doi: 10.3389/fonc.2023.1059914 37333805 PMC10272809

[18] Hleyhel M Bouvier AM Belot A Tattevin P Pacanowski J Genet P. Risk of non-AIDS-defining cancers among HIV-1-infected individuals in France between 1997 and 2009: results from a french cohort. AIDS. (2014) 28:2109–18. doi: 10.1097/QAD.0000000000000382 25265077

[19] Naidoo N Abayomi A Locketz C Musaigwa F Greiwal R. Incidence of Hodgkin lymphoma in HIV-positive and HIV-negative patients at a tertiary hospital in South Africa (2005-2016) and comparison with other African countries. S Afr Med J. (2018) 108:563–7. doi: 10.7196/SAMJ.2018.v108i7.12844 30004343

[20] Sung H Ferlay J Siegel RL Laversanne M Soerjomataram I Jemal A. Global cancer statistics 2020: GLOBOCAN estimates of incidence and mortality worldwide for 36 cancers in 185 countries. CA Cancer J Clin. (2021) 71:209–49. doi: 10.3322/caac.21660 33538338

[21] Huang J Pang WS Lok V Zhang L Lucero-Prisno DE III Xu W. Incidence, mortality, risk factors, and trends for Hodgkin lymphoma: a global data analysis. J Hematol Oncol. (2022) 15:57. doi: 10.1186/s13045-022-01281-9 35546241 PMC9097358

[22] Hu J Zhang X Tao H Jia Y. The prognostic value of Epstein–Barr virus infection in Hodgkin lymphoma: A systematic review and meta-analysis. Front Oncol. (2022) 12:1034398. doi: 10.3389/fonc.2022.1034398 36387159 PMC9648611

[23] Taj T Poulsen AH Ketzel M Geels C Brandt J Christensen JH. Long-term residential exposure to air pollution and Hodgkin lymphoma risk among adults in Denmark: a population-based case–control study. Cancer Causes Control. (2021) 32:935–42. doi: 10.1007/s10552-021-01446-w 34050843

[24] Hübel K Bower M Aurer I Bastos-Oreiro M Besson C Brunnberg U. Human immunodeficiency virus-associated Lymphomas: EHA–ESMO Clinical Practice Guideline for diagnosis, treatment and follow-up. HemaSphere. (2024) 8:e150. doi: 10.1002/hem3.150 39233903 PMC11369492

[25] Castelli R Schiavon R Preti C Ferraris L. HIV-related lymphoproliferative diseases in the era of combination antiretroviral therapy. Cardiovasc Hematol Disord Drug Targets. (2020) 20:175–80. doi: 10.2174/1871529X20666200415121009 PMC8226149 32294049

[26] Ferlay J Colombet M Soerjomataram I Parkin DM Pineros M Znaor A. Cancer statistics for the year 2020: an overview. Int J Cancer. (2021) 149:778–89. doi: 10.1002/ijc.33588 33818764

[27] Singh D Vacarella S Gini A Silva NDP Steliarova-Foucher E Bray F. Global patterns of Hodgkin lymphoma incidence and mortality in 2020 and a prediction of the future burden in 2040. Int J Cancer. (2022) 150:1941–7. doi: 10.1002/ijc.33948 35080783

[28] Pongas GN Ramos JC. HIV-associated lymphomas: progress and new challenges. J Clin Med. (2022) 11:1447. doi: 10.3390/jcm11051447 35268547 PMC8911067

[29] Marinescu AG Mardarescu M Vintila S Batan-Gherghe A Iosif I. Compartimentul pentru Monitorizarea şi Evaluarea Infecţiei HIV/SIDA în România – INBI “Prof.Dr.M.Balş”. In: Evoluția HIV în România – 31 Decembrie 2024. (Bucharest, Romania: Compartment for Monitoring and Evaluation of HIV/AIDS Infection in Romania)(2024). Available at: https://www.cnlas.ro/index.php/date-statistice.

[30] Arbune M Georgescu C Tutunaru D Dobre M Nechita A. Epidemiological aspects of new HIV/AIDS diagnoses in south-east Romania. BMC Infect Dis. (2014) 14:P53. doi: 10.1186/1471-2334-14-S2-P53

[31] Arbune M Debita M. Current issues on HIV adults in South East of Romania. Ro J Infect Dis. (2018) 21:1. doi: 10.37897/RJID.2018.1.2

[32] EACSociety. Available online at: https://www.eacsociety.org/media/2_neuropsychological_assessment_in_hivaids_and_its_challenges_in_galati:-_dr.marbune-_galati_Romania.pdf (Accessed December 14, 2024).

[33] Arbune M Padurariu-Covit MD Rebegea LF Lupasteanu G Arbune AA Stefanescu V. AIDS related kaposi's sarcoma: A 20-year experience in a clinic from the South-East of Romania. J Clin Med. (2021) 10:5346. doi: 10.3390/jcm10225346 34830628 PMC8620409

[34] World Health Organization. International Statistical Classification of Diseases and Related Health Problems 10th Revision. (Geneva, Switzerland: World Health Organization) (2019).

[35] Centers for Disease Control. 1993 revised classification system for HIV infection and expanded surveillance case definition for AIDS among adolescents and adults, (Atlanta: Morbidity and Mortality Weekly Report Recommendations and Reports (MMWR Recomm Rep)). Vol. 41. (1992). pp. 1–19. 1361652

[36] European AIDS Clinical Society Guidelines . Available online at: https://www.eacsociety.org/guidelines/guidelines-archive (Accessed October 10, 2024).

[37] Zelenetz AD Gordon LI Abramson JS Advani RH Andreadis B Bartlett NL. NCCN Guidelines® insights: B-cell lymphomas, version 6.2023: featured updates to the NCCN guidelines. J Natl Compr Canc Netw. (2023) 21:1118–31. doi: 10.6004/jnccn.2023.0057 37935098

[38] Gobbi PG Cavalli C Gendarini A Crema A Ricevuti G Federico M. Reevaluation of prognostic significance of symptoms in Hodgkin's disease. Cancer. (1985) 56:2874–80. doi: 10.1002/1097-0142(19851215)56:12<2874::AID-CNCR2820561227>3.0.CO;2-2 4052959

[39] Hoppe R Advani RH Ambinder RF Armand P Bello CM Beniez CM. Hodgkin Lymphoma Version 4.2024 — October 22, 2024. NCCN Clinical Practice Guidelines in Oncology (2024). Available online at: https://www.nccn.org/professionals/physician_gls/pdf/hodgkins.pdf (Accessed December 20, 2024).

[40] Leal VNC Reis EC Pontillo A. Inflammasome in HIV infection: lights and shadows. Mol Immunol. (2020) 118:9–18. doi: 10.1016/j.molimm.2019.12.001 31835091

[41] Cao D Khanal S Wang L Li Z Zhao J Nguyen LN. A matter of life or death: productively infected and bystander CD4 T cells in early HIV infection. Front Immunol. (2021) 11:626431. doi: 10.3389/fimmu.2020.626431 33643305 PMC7907524

[42] Lv T Cao W Li T. HIV-related immune activation and inflammation: current understanding and strategies. J Immunol Res. (2021) 1:7316456. doi: 10.1155/2021/7316456 PMC8494587 34631899

[43] Rice AP. The HIV-1 Tat protein: mechanism of action and target for HIV-1 cure strategies. Curr Pharm Design. (2017) 23:4098–102. doi: 10.2174/1381612823666170704130635 PMC5700838 28677507

[44] Isaguliants M Bayurova E Avdoshina D Kondrashova A Chiodi F Palefsky JM. Oncogenic effects of HIV-1 proteins, mechanisms behind. Cancers. (2021) 13:305. doi: 10.3390/cancers13020305 33467638 PMC7830613

[45] Okuma A Idahor C. Mechanism of increased cancer risk in HIV. Eur J Health Sci. (2020) 5:42–50. doi: 10.47672/ejhs.522

[46] Zhang F Bieniasz PD. HIV-1 Vpr induces cell cycle arrest and enhances viral gene expression by depleting CCDC137. eLife. (2020) 9:e55806. doi: 10.7554/eLife.55806 32538781 PMC7295576

[47] Bertuzzi C Sabattini E Bacci F Agostinelli C Ferri GG. Two different extranodal lymphomas in an HIV+ patient: a case report and review of the literature. Case Rep Hematol. (2019) 1:8959145. doi: 10.1155/2019/8959145 PMC6791282 31662919

[48] Ma W-L Liu W-D Sun H-Y Sheng W-H Hsieh S-M Wu S-J. Complete response to front-line therapies is associated with long-term survival in HIV-related lymphomas in Taiwan. J Microbiol Immunol Infect. (2024) 57:426–36. doi: 10.1016/j.jmii.2024.04.001 38632022

[49] Omar A Marques N Crawford N. Cancer and HIV: the molecular mechanisms of the deadly duo. Cancers (Basel). (2024) 16:546. doi: 10.3390/cancers16030546 38339297 PMC10854577

[50] Lurain K Ramaswami R Yarchoan R. The role of viruses in HIV-associated lymphomas. Semin Hematol. (2022) 59:183–91. doi: 10.1053/j.seminhematol.2022.11.002 PMC9971650 36805886

[51] Franceschi S Lise M Clifford G Rickenbach M Levi F Maspoli M. Changing patterns of cancer incidence in the early- and late-HAART periods: the Swiss HIV Cohort Study. Br J Cancer. (2010) 103:416–22. doi: 10.1038/sj.bjc.6605756 PMC2920013 20588274

[52] Long JL Engels EA Moore RD Gebo KA. Incidence and outcomes of Malignancy in the HAART era in an urban cohort of HIV-infected individuals. AIDS. (2008) 22:489–96. doi: 10.1097/QAD.0b013e3282f47082 PMC2553213 18301061

[53] Li CB Zhou Y Wang Y Liu S Wang W Lu X. In-hospital mortality and causes of death in people diagnosed with HIV in a general hospital in Shenyang, China: a cross-sectional study. Front Public Health. (2021) 9:774614. doi: 10.3389/fpubh.2021.774614 34917579 PMC8669430

[54] Phillips AA Smith DA. Health disparities and the global landscape of lymphoma care today. Am Soc Clin Oncol Educ Book. (2017) 37:226–33. doi: 10.1200/EDBK_175444 28561692

[55] Radkiewicz C Bruchfeld JB Weibull CE Jeppesen ML Frederiksen H Lambe M. Sex differences in lymphoma incidence and mortality by subtype: a population-based study. Am J Hematol. (2022) 98:23–30. doi: 10.1002/ajh.26744 36178436 PMC10092431

[56] Pfreundschuh M. Age and sex in non-Hodgkin lymphoma therapy: it's not all created equal, or is it? Am Soc Clin Oncol Educ Book. (2017) 37:505–11. doi: 10.1200/EDBK_175447 28561693

[57] Vargas JC Marques MO Pereira J Braga WMT Hamerschlak N Tabacof J. Factors associated with survival in patients with lymphoma and HIV. AIDS. (2023) 37:1217–26. doi: 10.1097/QAD.0000000000003549 36939075

[58] Wu D Chen C Zhang M Li Z Wang S Shi J. The clinical features and prognosis of 100 AIDS-related lymphoma cases. Sci Rep. (2019) 9:5381. doi: 10.1038/s41598-019-41869-9 30926889 PMC6441082

[59] Vandenhende MA Roussillon C Henard S Morlat P Oksenhendler E Aumaitre H. Cancer-related causes of death among HIV-infected patients in France in 2010: evolution since 2000. PloS One. (2015) 10:e0129550. doi: 10.1371/journal.pone.0129550 26083524 PMC4470800

[60] Shiels MS Pfeiffer RM Gail MH Hall HI Li J Chaturvedi AK. Cancer burden in the HIV-infected population in the United States. J Natl Cancer Institute. (2011) 103:753–62. doi: 10.1093/jnci/djr076 PMC3086877 21483021

[61] Moahi K Ralefala T Nkele I Triedman S Sohani A Musimar Z. HIV and hodgkin lymphoma survival: A prospective study in Botswana. JCO Global Oncol. (2022) 8:e2100163. doi: 10.1200/GO.21.00163 PMC8769145 35025689

[62] WHO Regional Office for Europe European Centre for Disease Prevention and Control. HIV/AIDS surveillance in Europe 2024–2023 data. Copenhagen: WHO Regional Office for Europe (2024). Available at: https://www.ecdc.europa.eu/en/publications-data/hiv-aids-surveillance-europe-2024-2023-data (Accessed January 3, 2025).

[63] Mardarescu M. Future directions in HIV in relation to the four”95”. 2024 ECDC Network meeting, 15–17 April 2024. (Stockholm, Sweden: European Centre for Disease Prevention and Control (ECDC)) (2024). Available at: https://www.cnlas.ro/images/doc/2024/HIV%20Future%20Directions.pdf.

[64] Ministry of Health. National Institute of Infectious Diseases “Prof. Dr. Matei Balş” Compartment for Monitoring and Evaluation of HIV/AIDS Infection in Romania. Statistic data (2003, 2012, 2024). Available online at: https://www.cnlas.ro/index.php/date-statistice (Accessed December 24, 2024).

[65] Preda M Manolescu LC. Romania, a harbour of HIV-1 subtype F1: where are we after 33 years of HIV-1 infection? Viruses. (2022) 14:2081. doi: 10.3390/v14092081 36146886 PMC9503723

[66] Arbune M Georgescu CV Voinescu DC. AIDS-related lymphoma in a young HIV late presenter patient. Romanian J Morphology Embryology. (2016) 57:273–6. 27151720

[67] Oseso LN Chiao EY Ignacio RAB. Evaluating antiretroviral therapy initiation in HIV-associated Malignancy: Is there enough evidence to inform clinical guidelines? J Natl Compr Canc Netw. (2018) 16:927–32. doi: 10.6004/jnccn.2018.7057 PMC6207434 30099368

[68] Sereti I. Immune reconstruction inflammatory syndrome in HIV infection: Beyond what meets the eye. Top Antivir Med. (2020) 27:106–11. PMC7162680 32224502

[69] Vargas JC Cecyn KZ Oliveira Marques M Pereira J Tobias Braga WM Hamerschlak N. Immune reconstitution inflammatory syndrome-associated lymphoma: A retrospective Brazilian cohort. EJHaem. (2023) 5:147–52. doi: 10.1002/jha2.835 PMC10887249 38406522

[70] Gopal S Patel MR Achenbach CJ Yanik EL Cole SR Napravnik S. Lymphoma immune reconstitution inflammatory syndrome in the center for AIDS research network of integrated clinical systems cohort. Clin Infect Dis. (2014) 59:279–86. doi: 10.1093/cid/ciu270 PMC4102912 24755860

[71] McNally GA. HIV and cancer: An overview of AIDS-defining and non–AIDS-defining cancers in patients with HIV. Clin J Oncol Nurs. (2019) 23:327–31. doi: 10.1188/19.CJON.327-331 31099792

[72] Uldrick TS Ison G Rudek MA Noy A Schwartz K Bruinooge S. Modernizing clinical trial eligibility criteria: Recommendations of the American Society of Clinical Oncology-Friends of Cancer Research HIV Working Group. J Clin Oncol. (2017) 35:3774–80. doi: 10.1200/JCO.2017.73.7338 PMC5793223 28968173

[73] Vora KB Ricciuti B Awad MM. Exclusion of patients living with HIV from cancer immune checkpoint inhibitor trials. Sci Rep. (2021) 11:6637. doi: 10.1038/s41598-021-86081-w 33758321 PMC7988004

[74] Carbone A Vaccher E Gloghini A. Hematologic cancers in individuals infected by HIV. Blood. (2022) 139:995–1012. doi: 10.1182/blood.2020005469 34469512

[75] Wang C Wu Y Liu J Min H Huang Y Wei G. Impact of initial chemotherapy cycles and clinical characteristics on outcomes for HIV-associated diffuse large B cell lymphoma patients: The Central and Western China AIDS Lymphoma League 001 study (CALL-001 study). Front Immunol. (2023) 14:1153790. doi: 10.3389/fimmu.2023.1153790 37063928 PMC10090414

[76] Hernández-Ramírez RU Qin L Lin H Leyden W Neugebauer RS Althoff KN. North American AIDS Cohort Collaboration on Research and Design of the International Epidemiologic Databases to Evaluate AIDS Collaborators. Association of immunosuppression and HIV viraemia with non-Hodgkin lymphoma risk overall and by subtype in people living with HIV in Canada and the USA: a multicentre cohort study. Lancet HIV. (2019) 6:e240–9. doi: 10.1016/S2352-3018(18)30360-6 PMC6531288 30826282

[77] Re A Cattaneo C Montoto S. Treatment management of haematological Malignancies in people living with HIV. Lancet Haematol. (2020) 7:e679–89. doi: 10.1016/S2352-3026(20)30115-0 32791044

[78] Bertoli D Re A Chiarini M Sottini A Serana F Giustini V. B-and T-lymphocyte number and function in HIV+/HIV– lymphoma patients treated with high-dose chemotherapy and autologous bone marrow transplantation. Sci Rep. (2016) 6:37995. doi: 10.1038/srep37995 27905485 PMC5131356

[79] Abbasi A Peeke S Shah N Mustafa J Khatun F Lombardo A. Axicabtagene ciloleucel CD19 CAR-T cell therapy results in high rates of systemic and neurologic remissions in ten patients with refractory large B-cell lymphoma including two with HIV and viral hepatitis. J Hematol Oncol. (2020) 13:1–4. doi: 10.1186/s13045-019-0838-y 31900191 PMC6942268

